# Denture Wearing and Malnutrition Risk Among Community-Dwelling Older Adults

**DOI:** 10.3390/nu12010151

**Published:** 2020-01-05

**Authors:** Ya Su, Michiko Yuki, Kengo Hirayama, Miho Sato, Tianfang Han

**Affiliations:** 1 Graduate School of Health Sciences, Hokkaido University, Sapporo 060-0812, Japan; swanvivi@eis.hokudai.ac.jp (Y.S.); htf@eis.hokudai.ac.jp (T.H.); 2 Faculty of Health Sciences, Hokkaido University, Sapporo 060-0812, Japan; khirayama@hs.hokudai.ac.jp (K.H.); m_sato@med.hokudai.ac.jp (M.S.)

**Keywords:** malnutrition, denture wearing, salt taste, community dwelling

## Abstract

Most studies have associated denture wearing with a higher risk of malnutrition and lower nutritional intake in the elderly. As the effect of wearing dentures remains debatable, the aim of this study was to determine if older adults wearing dentures have a higher risk of malnutrition. We recruited 294 Japanese community-dwelling adults aged ≥65 years from two elderly welfare centers in Sapporo. The Mini Nutritional Assessment-Short Form (MNA-SF) was used to assess their nutritional status. Their demographic characteristics, eating habits, and health status were assessed using a questionnaire. Moreover, salt-impregnated taste paper and bioelectrical impedance analysis were used to obtain salt taste sensitivity and body composition (body mass index and protein mass). The mean age of older adults was 75.8 ± 5.8 years and 71.1% were women. We found that 74.1% of the older adults wore dentures. The proportion of the whole population with a risk of malnutrition was 23.5%. Multivariate analysis revealed that partial denture wearing could be protective against the risk of malnutrition. In this study, we found more partial denture wearers with normal nutritional conditions and cooking by themselves than non-denture-wearing older adults. Furthermore, complete denture wearers were eating significantly less meats or fish every day. This study suggests that using partial dentures to replace the lost teeth to meet the dietary needs of the elderly is critical to maintaining a healthy nutritional status. Moreover, it also indicates the importance of providing education to older adults who wear complete dentures.

## 1. Introduction

The average life expectancy in adults is increasing worldwide. These changes have resulted in increasing numbers of older populations aged 60 years or more. It is estimated that by the year 2030 older people will outnumber younger people as the number of people in the world aged over 60 years is projected to grow by 56% from 2015 to 2030 [1]. In Japan, the proportion of the population who were aged 65 years or over was 27.6% in 2017, and this statistic is estimated to exceed 38.4% in 2065 [2]. Older adults tend to be more prone to malnutrition which is associated with an increased risk of morbidity and mortality [3] due to problems such as increased falls, function decline, frailty, and poor quality of life [4,5]. Achieving and maintaining a good nutritional status has significant implications for health outcomes, and for promoting functional and living independence. Aging is accompanied by many changes that make it more difficult to maintain nutrition. Factors such as bereavement or social isolation can influence dietary practices [6]. Moreover, older adults with chronic diseases such as respiratory disease, stroke, and dementia have a higher risk of malnutrition [7]. Among these diseases and complications, stroke and dementia reflect high rates of dysphagia, which often results in malnutrition and increased risk of pneumonia [8]. Furthermore, mental health problems such as depression and anxiety may also increase the risk of malnutrition [9].

In additional, oral health is one of the conditions that may influence nutritional status, such as taste sensitivity, dry mouth, periodontal disease, and dental status [7]. According to studies, one in five older people reported that an oral condition prevented them from eating the foods they would choose [10]. The effect of dentures on diet and nutritional status among older adults has been documented [11]. A Tufts University study showed that denture wearers intake lower nutrients than older adults with more of their teeth. Many of the nutrients missing are found in hard-to-chew foods such as stringy meats, and certain vegetables and fruits [12]. A previous study has also shown that people with <20 teeth or wearing complete dentures had lower nutritional intakes than people with more than 20 teeth [13]. Normally an adult mouth has 32 teeth in total, but millions of people around the world have lost all their teeth. The mean number of remaining natural teeth is lower than 20 in most countries, particularly in those over 75 years of age [14]. Most studies have shown an association of tooth loss and denture-wearing with lower nutritional intake and higher malnutritional risk [11,15]. A systematic review and meta-analysis investigating the association between oral health in individuals older than 60 years and nutritional status, however, found no associations between dental status and malnutrition [16]. A previous study has reported that people with self-perceived good fitting dentures had diets that were not different from participants with 18 or more teeth, indicating that successful dental treatment may improve dietary selection [17]. These findings are not consistent, and most studies focused on complete dentures. When only some teeth are missing, the effect of using partial dentures remains unknown. Thus, this study is to determine if older adults wearing complete or partial dentures have a higher risk of malnutrition, and additionally, to compare the related factors between these states.

## 2. Materials and Methods

### 2.1. Study Design

Participants were drawn from the Community-Based Aging and Nutrition Study (CBAN Study). This study is a cross-sectional study that aims to investigate the nutritional conditions of Japanese community-dwelling older people in order to improve their nutrition, functional performance, and quality of life.

### 2.2. Study Population

The study population was recruited from regular attendees of two elderly welfare centers in August and September 2018 in Sapporo, Japan. The elderly welfare center is a facility that provides health promotion and recreation for community-dwelling older adults aged 60 and over, such as physical exercise, recreational activities, and health lectures. It also organizes events to deepen communication with community-dwelling older people. Participants who met the following criteria were included: (1) aged 65 years or older; (2) able to walk without help; (3) willing to complete the survey; and (4) provided consent to participate.

### 2.3. Ethics Approval and Consent to Participate

This study was approved by the Ethics Committee of the Faculty of Health Sciences, Hokkaido University (Reference No 18-22-1). Each participant signed an informed consent document after receiving a detailed explanation of the study objectives.

### 2.4. Variables and Measuring Instruments

Age, gender, living alone, smoking status, alcohol consumption, eating habits, and health status (nutritional status, protein mass, dental status, salt taste sensitivity, dysphagia, depression status, frailty, chronic diseases, and prescription drug use) were assessed using questionnaires and measurements.

#### 2.4.1. Eating Habits

The questionnaire on eating habits includes seven items concerning eating alone, self-cooking, awareness of salt restriction, and consuming dairy products, legumes, meats, fish, fruit or vegetables, which were as follows: (1) Who do you usually eat with? (2) Do you cook your own meals? (3) Do you moderate salt intake when eating? (4) Do you consume at least one serving of dairy products (milk, cheese, yogurt, etc.) every day? (5) Do you consume two or more servings of legumes or eggs every week? (6) Do you eat meats or fish every day? (7) Do you consume two or more servings of fruits or vegetables every day?

#### 2.4.2. Nutritional Status

To assess the nutritional status of the participants, the Mini Nutritional Assessment Short Form (MNA^®^-SF) was used; it comprises six questions, including the decline of food intake, weight loss in the last three months, mobility, actual disease/distress in the last three months, neuropsychological problems, and additional anthropometric measures (Body Mass Index: BMI) [18]. The MNA^®^-SF has been shown to be a valid nutrition screening tool for use in community-dwelling older adults [19]. The scores of the short-form MNA^®^ are summed to give a total of 0–14; In this study, scores of <12 were considered to indicate a malnutrition risk [18].

#### 2.4.3. Protein Mass and Body Mass Index

Protein mass and BMI were measured using bioelectrical impedance analysis (BIA) (InBodyS10, Biospace, Seoul, Korea) with a seated posture for participants who have no pacemaker implantation. The participants sat and rested for approximately 10–15 min before the test, arms were posed and naturally lowered, and thighs did not touch each other but were spread to shoulder width.

#### 2.4.4. Dental Status

Dental status included complete denture wearing (CDW), partial denture wearing (PDW), and non-denture wearing (NDW) for which they self-reported, and the number of present teeth were counted by researchers. The definition of complete denture (CD) were participants who used a removable appliance when all teeth within a jaw had been lost. Partial denture (PD) were those who used dentures when only some teeth were missing. Non-denture wearing (NDW) were those who did not use dentures even with some missing teeth.

#### 2.4.5. Salt Taste Sensitivity

For the assessment of the gustatory threshold for salty taste, a salt-impregnated taste paper, Salsave, was used (Advantec Tokyo Co. Ltd., Tokyo, Japan), which is impregnated with various salt concentrations (0.6, 0.8, 1.0, 1.2, 1.4, and 1.6 mg/cm^2^); results at a concentration of ≤1.0 mg/cm^2^ were considered as taste impaired [20,21]. The assessment was performed by researchers. First, the participants were asked to rinse their mouths with distilled water to neutralize oral conditions as much as possible. Then they were asked to place a strip saturated with salt chloride on their tongue for 3 s. The lowest concentration at which participants identified the salt taste was taken as the recognition threshold. Participants who could not recognize taste, even at the highest concentration level (1.6 mg/cm^2^), were assigned 1.8 [22].

#### 2.4.6. Dysphagia Screening

The dysphagia screening used the Seirei dysphagia questionnaire which consists of 15 items including history of pneumonia, nutritional status, pharyngeal, oral, and esophageal phases, and glottic structures. The answers are A (severe and frequent symptoms), B (mild and infrequent symptoms), and C (no symptoms). Dysphagia is suspected when the answers have a single A. The questionnaire shown high reliability, sensitivity, and specificity, and is easy to administer and useful in diverse populations [23,24,25].

#### 2.4.7. Depression Screening

The 15-item Geriatric Depression Scale (GDS-15) was used to assess depression states among the participants. This is a short version developed in 1986 which consists of 15 questions. Questions from the Long-Form GDS, which had the highest correlation with depressive symptoms in validation studies, were selected and the total scores are in the range 0–15; scores of >5 were considered to indicate depression [26]. The GDS-15 is a reliable and valid screening tool for major depression in community-living Asian older adults [27], although it is not a substitute for a diagnosis by mental health professionals. It is a useful screening tool to advance depression assessment in older adults [28].

#### 2.4.8. Frailty Screening

We assessed the frailty phenotype using the frailty screening index, which was based on the Cardiovascular Health Study criteria, the Kihon checklist, and other Japanese questionnaires [29]. The frailty screening index includes five items concerning weight loss, physical function, physical activity, memory loss, and exhaustion, which were as follows: (1) “Have you lost 2 kg or more in the past 6 months?”, (2) “Do you think you walk slower than before?”, (3) “Do you go for a walk for your health at least once a week?”, (4) “Can you recall what happened 5 min ago?”, and (5) “In the past 2 weeks, have you felt tired without a reason?” The total ranges from 0–5; scores of ≥3 were considered frail according to the Cardiovascular Health Study criteria. This frailty screening index is a useful self-report questionnaire for frailty in community-dwelling older adults [29,30]. 

### 2.5. Statistical Analyses

All data analyses were conducted using IBM SPSS Statistics Version 22.0 (IBM, Armonk, New York, NY, USA). Statistical analyses included univariate and multivariate analysis. Continuous variables were presented using mean and standard deviations, and categorical variables were presented using frequencies and percentages. The continuous variables with a normal distribution were used to perform Student’s *t*-test, a one-way ANOVA, and the non-parametric Mann–Whitney U-test was used for the variables with a non-normal distribution. Additionally, post-hoc analysis, including the chi-squared test, Fisher’s exact test, and chi-square partition were used with the categorical variables. Finally, a binary logistic regression analysis was performed to determine the association of denture wearing and malnutrition risk. *p* values < 0.05 were considered statistically significant.

## 3. Results

We recruited 310 participants from regular attendees of two centers in Sapporo. A total of 294 community-dwelling older adults completed the questionnaire and measurements with a response rate of 95%. The mean age of participants was 75.8 ± 5.8 years, and 71.1% (209) of participants were women. There were 218 (74.1%) of the older adults who wore dentures; 9.9% were CD wearers and 64.3% were PD wearers. The average numbers of teeth was 24.1 ± 6.2 teeth and 14.3 ± 7.7 teeth in the ND wearers and PD wearers, respectively. Furthermore, there were 69 (23.5%) older adults at risk of malnutrition according to the screening (MNA-SF < 12) in the entire study population. The mean MNA-SF scores were 12.4 ± 1.8.

**Figure 1:**
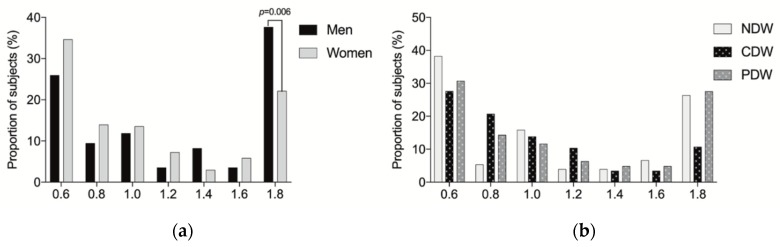
The percentage of the subjects with each taste recognition threshold for salt. (**a**) The percentage of the subjects with each taste recognition threshold for salt by sex; (**b**) The percentage of the subjects with each taste recognition threshold for salt by dental status. NDW: Non denture wearing; CDW: Complete denture wearing; PDW: Partial denture wearing.

The characteristics of participants with gender are shown in Table 1. There was no significant difference in nutritional status and denture wearing between men and women. However, more women than men lived and ate alone. Additionally, men were older than women. Not surprisingly, smoking, consuming alcohol, BMI, and protein mass were significantly lower in women than in men. Additionally, self-cooking was significantly higher in women than in men, and more women were eating at least one serving of dairy products every day. There were 55 (18.7%) participants found to have suspected dysphagia, with more men than women found to have suspected dysphagia. We found 46 (15.6%) participants who were considered depressed (GDS-15 > 5) in the whole study population. The mean GDS-15 scores were 3.3 ± 2.6. The prevalence of frailty was 9.5% (28) according to the screening. There was no difference in depression and frailty between men and women. We found 162 (55.1%) participants to be taste impaired according to the salt-impregnated taste paper; this was significantly higher in men than in women. As shown in Figure 1, the percentage of the subjects with each taste recognition threshold for salt (0.6, 0.8, 1.0, 1.2, 1.4, 1.6, and 1.8 mg/cm^2^) was, 25.9%, 9.4%, 11.8%, 3.5%, 8.2%, 3.5%, and 37.6% among men, and 34.6%, 13.9%, 13.5%, 7.2%, 2.9%, 5.8%, and 22.1% among women, respectively. Men showed the higher percentage of recognition threshold for salt at 1.8 than women. This level was for participants who could not recognize the taste even at the highest concentration level (1.6 mg/cm^2^).

**Figure 2:**
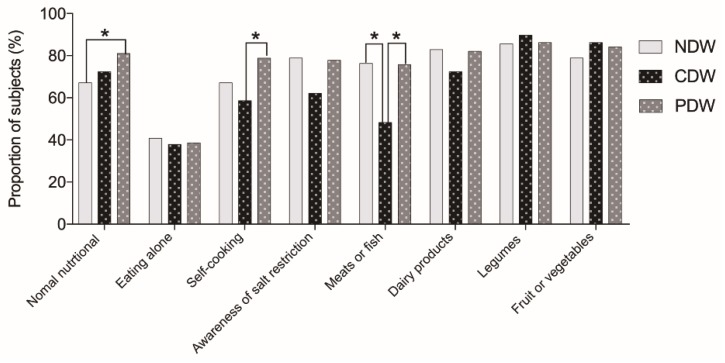
The percentage of the subjects with normal nutritional and eating habits by dental status. *: *p* < 0.017.

Characteristics of participants for malnutrition risk in univariate analysis are shown in Table 2. The variables significantly associated with malnutrition risk were wearing dentures, suspected dysphagia, frailty, protein mass, and awareness of salt restriction. As shown in Figure 2, more PD wearers than ND older adults were at normal nutrition. Variables independently associated with malnutrition risk in multivariate analysis are shown in Table 3. After adjusting for age, gender, suspected dysphagia, frailty, protein mass, and awareness of salt restriction, wearing PD was found to be independently negatively associated with malnutrition risk (OR, 0.39, 95% CI, 0.20–0.77).

Eating habits associated with denture wearing are shown in Figure 1b and Figure 2. There was no significant difference between denture wearing and salt taste sensitivity or eating fruits and vegetables every day. However, CD wearers ate significantly less meats or fish every day. PD wearers were more likely to cook by themselves.

## 4. Discussion

Malnutrition, a common health problem in the elderly, leads to poor outcomes such as functional decline, frailty, declining quality of life, and higher mortality [4,5]. Around the world, tooth loss is seen as being in direct relation to aging [31]. Building upon few studies that have investigated the relationship between partial dentures and nutritional status and lifestyle, this study has provided evidence to further elucidate this topic. In the present cross-sectional study of community-dwelling Japanese older adults, we investigated the relationship between denture-wearing status and malnutrition risk, and we found that partial denture wearing could protect against the risk of malnutrition. Moreover, complete denture wearers were eating significantly less meats or fish every day.

Previous studies showed that poor dental status can result in malnutrition caused by eating less food due to difficulty chewing [10,32]. Masticatory efficiency is affected by the presence of teeth and the use of dentures, which influence the choice of food [33]. In this study, the average number of present teeth was 15.5 ± 9.6 in the whole of the study population. There were 74.1% of the older adults who wore dentures. The results of the study were consistent with previous studies in that the average number of present teeth was 18.1 ± 7.6 in men, and 17.4 ± 7.9 in women [34]. Additionally, a prevalence of 23.7% for the risk of malnutrition was observed within the population. The results were consistent with previous studies that showed that the prevalence of malnutrition risk was 22%–34.7% [35,36,37].

Malnutrition among older adults is influenced by many factors. Most studies have shown dysphagia is associated with malnutrition. Dysphagia refers to difficulty swallowing that can reduce or alter food intake and may result in malnutrition [38]. Moreover, nutritional status has been identified as a key factor in preventing the development of frailty syndrome. A community-based study reported an increased risk of frailty with low energy intake and inadequate intake of protein, vitamin D, vitamin E, vitamin C, and vitamin B9 [39]. The results of this study are consistent with previous studies. Most studies have shown that malnutrition was independently associated with depression [40]. Furthermore, depression leads to loss of appetite and consequently weight loss. However, a community-based study reported 21.5% of older adults with depression, and there was an association between GDS and MNA-SF for young-old individuals (aged 65-74 years), but not for old-old individuals (aged 75 years and over) or for the two groups combined [41]. In this study, 15.7% of participants were considered to have depression and there was no significant difference between malnutritional risk and depression. Perhaps this is because most of the participants are regular attendees in the welfare center with active social participation and exercise.

In this study, the low protein mass measured by BIA was significantly associated with malnutrition risk assessed by the MNA-SF, however, we found no significate difference between protein intake (dairy products, legumes, or egg, meats, or fish) and malnutrition risk. Most studies reported that denture wearers can be at risk of malnutrition [10,11,16]. A study has shown that complete or partial denture wearers had diets considerably lower in 19 different nutrients compared to adults with more of their teeth [12]. Another study has shown the edentulous participants and denture wearers had a significantly lower intake of vitamin A than the referent population [38]. However, most previous studies found there were no associations between dental status and nutritional status among older adults [16,42,43] In this study, we also did not find an association between complete denture wearing and malnutrition risk, but the most important finding is the partial denture-wearing benefit for nutritional status among older adults. In this study, the average number of teeth was 24.1 ± 6.2 in the NDW older adults. It seems like some NDW older adults lost their teeth but did not replace them. Considering that replacing lost teeth with partial dentures would improve chewing, this study found more partial denture wearers cooking by themselves, which suggests these older people may cook foods that are more appropriate for them to chew according to their dental status, and that this decreases their malnutritional risk.

Previous studies have shown that multiple reasons may affect an individual’s experience in wearing their dentures. One of the reasons is the minimization of taste and texture sensation due to the covering of the palate [15]. Taste changes that occur with advancing age are associated with nutritional status. It can lead to poor appetite that changes food choices and lower nutrient intake, and may result in weight loss or malnutrition [44]. Taste sensitivity may be reduced by complete denture wearing [10]. However, in this study, we found no association between salt taste sensitivity and dental status. We found that those with no awareness of salt restriction were associated with higher malnutrition risk. Denture wearing with reduced chewing ability can also affect the diet. A previous study reported participants with intact dentitions chewed the best, followed by partial denture wearers, and the last were participants who wore complete dentures [45]. A study has shown that partial and complete denture wearers had lower nutrient intakes than older adults with more of their teeth. Many of the nutrients missing are found in hard-to-chew foods such as stringy meats, some vegetables, and fruits [12]. We found eating fruits or vegetables was not associated with nutritional status. However, the complete denture wearers were eating significantly less meats or fish every day. A previous study reported that decreased numbers of functional teeth are associated with the avoidance of stringy meats [12,46]. Meats, fish, and milk contributed the most to protein intake in the older population [47]. Inadequate consumption of protein has shown associated with poor muscle function and physical decline in older adults [48]. It has been reported that older adults need greater doses of protein (beef) to enhance the rate of postprandial muscle protein synthesis [49]. There are many evidences shown that inadequate intake of fish could impact on the cognitive function and increase the risk of cardiovascular disease [50,51,52]. Replacing lost teeth and meeting the dietary and nutritional needs of the elderly is critical to maintaining health and quality of life. However, there are some limitations to this study. First, CBAN Study is a cross-sectional study; therefore, it cannot prove causality between wearing dentures and malnutritional risk. Second, the relatively small sample size of this study, particularly the size of the complete denture wearers, is small. Studies with larger sample sizes are needed to confirm these findings. Third, we did not confirm the fit and comfort of dentures as dentures may need alterations over time. Finally, nutritional supplements have been shown to have a positive effect on nutritional status, and we did not confirm whether the participants took nutritional supplements.

Despite many older people losing their teeth, not everyone will seek treatment to repair and replace them. The World Health Survey which was attended by 72 countries has reported that loss of teeth is lower in older people of the African and Southeast Asia regions. In several high-income countries, older people often had their teeth extracted because of pain or discomfort. Loss of teeth without denture replacement means severe loss of oral function, and this situation is common among poor populations in both developed and developing countries. In addition, many reports indicated that the demand for denture treatment and dental restorations has not been met, particularly among the older population [53]. Researchers and nutritionists often explore secondary risk factors associated with malnutrition and improve diets for older people through health promotion education. However, they have paid relatively little attention to teeth, which is one of the fundamental and often overlooked factors inhibiting eating and can affect food choice. The types and usage of dentures are related to appetite, diet quality, food choices, and intake of some nutrients. Therefore, a better understanding of the eating habits and impact of older people with dentures may provide tailored guidance for those at risk.

## 5. Conclusions

The present study had limitations, and future longitudinal studies are necessary to verify the findings from this study. Despite these limitations, the present study has important implications for health professionals and future studies. The study demonstrated the relationship between partial denture wearing and nutritional conditions. Moreover, complete denture wearers were eating significantly less meats or fish every day. This study suggests that using partial dentures to replace the lost teeth and meeting the dietary needs of the elderly is critical to maintaining nutritional status. It also indicates the importance of providing education to the community-dwelling older adults who wear complete dentures.

## Figures and Tables

**Table 1:** Characteristics of participants by sex.

Variables	Men n = 85	Women n = 209	*p* Value
Age (years)	77.2 ± 5.7	75.2 ± 5.8	0.008 **
Living alone, n (%)	17 (20.0)	84 (40.2)	0.001 **
Smoking, n (%)			<0.001 **
Current	12 (14.1)	10 (4.8)	
Former	46 (54.1)	22 (10.5)	
Consumes alcohol, n (%)	47 (55.3)	66 (31.6)	<0.001 **
Health status			
Diabetes, n (%)	8 (9.4)	16 (7.7)	0.618
Hypertension, n (%)	20 (23.5)	52 (24.9)	0.807
Take >4 prescription drugs per day, n (%)	31 (36.5)	59 (28.2)	0.165
Suspected dysphagia, n (%)	26 (30.6)	29 (13.9)	0.001 *
Depression, n (%)	14 (16.5)	32 (15.3)	0.804
Frailty, n (%)	12 (14.1)	16 (7.7)	0.087
BMI	23.4 ± 3.1	22.5 ± 2.8	0.011 *
Protein mass (kg)	9.4 ± 1.1	6.9 ± 0.7	<0.001 **
Dental status, n (%)			0.205
Complete denture	8 (9.4)	21 (10.0)	
Partial denture	49 (57.6)	140 (67.0)	
Eating habits, n (%)			
Eating alone	21 (24.7)	94 (45.0)	0.001 **
Self-cooking	19 (22.4)	198 (94.7)	<0.001 **
Awareness of salt restriction	51 (60.0)	174 (83.3)	<0.001 **
Salt taste impaired	55 (64.7)	107 (51.2)	0.035 *
At least one serving of dairy products per day	63 (74.1)	176 (84.2)	0.044 *
Two or more servings of legumes per week	73 (85.9)	181 (86.6)	0.870
Meats or fish every day	55 (64.7)	160 (76.6)	0.038
Two or more servings of fruit or vegetables per day	69 (81.2)	75 (83.7)	0.597

Results are presented as mean ± SD, or n (%); *: *p* < 0.05; **: *p* < 0.01.

**Table 2:** Characteristics of participants with nutritional status.

Variables	Total n = 294	Normal Nutritional n = 225	Malnutrition Risk n = 69	*p* Value
Age (years)	75.8 ± 5.8	75.9 ± 5.7	75.3 ± 6.3	0.456
Female, n (%)	209 (71.1)	154 (68.4)	55 (79.7)	0.071
Living alone, n (%)	101 (34.4)	73 (32.4)	28 (40.6)	0.213
Smoking, n (%)				0.134
Current	22 (7.5)	13 (5.8)	9 (13.0)	
Former	68 (23.1)	53 (23.6)	15 (21.7)	
Consumes alcohol, n (%)	113 (38.4)	89 (39.6)	24 (34.8)	0.476
Health status				
Diabetes, n (%)	24 (8.2)	19 (8.4)	5 (7.2)	0.751
Hypertension, n (%)	72 (24.5)	57 (25.3)	15 (21.7)	0.544
Take >4 prescription drugs per day, n (%)	90 (30.6)	70 (31.1)	20 (29.0)	0.738
Suspected dysphagia, n (%)	55 (18.7)	36 (16.0)	19 (27.5)	0.032 *
Depression, n (%)	46 (15.6)	31 (13.8)	15 (21.7)	0.111
Frailty, n (%)	28 (9.5)	16 (7.1)	12 (17.4)	0.011 *
Protein mass (kg)	7.6 ± 1.4	7.8 ± 1.5	7.0 ± 1.1	<0.001 **
Dental status, n (%)				0.048 *
Complete denture	29 (9.9)	21 (9.3)	8 (11.6)	
Partial denture	189 (64.3)	153 (68.0)	36 (52.2)	
Eating habits, n (%)				
Eating alone	115 (39.1)	84 (37.3)	31 (44.9)	0.258
Self-cooking	217 (73.8)	165 (73.3)	52 (75.4)	0.737
Awareness of salt restriction	225 (76.5)	179 (79.6)	46 (66.7)	0.027 *
Salt taste impaired	162 (55.1)	124 (55.1)	38 (55.1)	0.995
At least one serving of dairy products per day	239 (81.3)	185 (82.2)	54 (78.3)	0.460
Two or more servings of legumes per week	254 (86.4)	198 (88.0)	56 (81.2)	0.147
Meats or fish every day	215 (73.1)	169 (75.1)	46 (66.7)	0.166
Two or more servings of fruit or vegetables per day	244 (83.0)	181 (80.4)	63 (91.3)	0.036 *

Results are presented as mean ± SD, or n (%); *: *p* < 0.05, **: *p* < 0.01.

**Table 3:** Association between dental status and malnutrition risk in logistic regression analysis.

Variables	B	SE	OR (95% CI)	*p* Value
Age	−0.045	0.029	0.96 (0.90–1.01)	0.118
Female	−0.679	0.587	0.51 (0.16–1.60)	0.247
Dental status				0.024
Complete denture	−0.463	0.546	0.63 (0.22–1.83)	0.396
Partial denture	−0.945	0.348	0.39 (0.20–0.77)	0.007 **
Awareness of salt restriction	−1.055	0.365	0.35 (0.17–0.71)	0.004
Suspected dysphagia	1.171	0.388	3.23 (1.51–6.90)	0.003
Frailty	1.107	0.475	3.03 (1.19–7.68)	0.020
Protein mass	−0.895	0.209	0.41 (0.27–0.62)	<0.001

B: coefficient of logistic regression; OR: odds ratio; CI: confidence interval; Model Fitting criteria: The Hosmer–Lemeshow test for logistic regression gave *p* = 0.467; Age, gender, suspected dysphagia, frailty, protein mass, and awareness of salt restriction were adjusted for; **: *p* < 0.01.
